# The interdisciplinary implementation of poly-universe to promote computational thinking: Teaching examples from biological, physical, and digital education in Austrian secondary schools

**DOI:** 10.3389/fpsyg.2023.1139884

**Published:** 2023-03-15

**Authors:** Eva Schmidthaler, Maritta Schalk, Mathias Schmollmüller, Sara Hinterplattner, Corinna Hörmann, Branko Anđić, Marina Rottenhofer, Zsolt Lavicza, Barbara Sabitzer

**Affiliations:** ^1^Johannes Kepler University, School of Education, Linz, Austria; ^2^Dynatrace, Linz, Austria

**Keywords:** poly-universe, computational thinking, digital education, game-based learning, biology, physical education, stem

## Abstract

Today’s teaching and didactical methods are progressively aiming to integrate digital technologies, computational thinking (CT), and basic computer science concepts into other subjects. An innovative and creative way of combining and integrating CT and teaching cross-curricular skills without digital devices is to include the game Poly-Universe (PolyUni). According to previous research, the game is expected to have a positive effect on visual perceptual progress, including isolation, and the development of shape-background skills. So far, however, comparatively few attempts have been made to explore the educational possibilities of PolyUni for different school levels and subjects, besides mathematics. Therefore, this article aims to close this gap by exploring how PolyUni can be used to promote CT in three subjects: physical education (PE), digital education (DGE), and biology (B). Furthermore, it evaluates whether the pre-defined learning objectives in those subjects have been achieved, and examines how PolyUni combines the requirements of the different curricula in Austrian secondary school, based on self-designed tasks. Additionally, further aspects of PolyUni such as engagement and collaboration are discussed. To explore the above-mentioned benefits, a mixed-methods study was implemented, whereas the workshops and accompanying teaching materials (e.g., worksheets) were developed based on the COOL Informatics concept. The participant observation method was employed for qualitative data collection, and a self-designed assessment grid as well as additional picture analysis were used for the quantitative data. PolyUni was introduced in three different workshops at Austrian secondary schools with 80 students observed and analyzed. Based on the present data, it can be assumed that PolyUni supports achieving the requirements of the different curricula and pre-defined teaching and learning objectives in a playful way. Furthermore, the game not only promotes CT in secondary school but also encourages enjoyment and collaboration between peers in biological, digital, and physical education lessons.

## 1. Introduction

Computational thinking (CT) is a term that has become more and more present in the last years and decades, especially since Jeanette Wings used the expression in 2006 (Selby and Woollard, 2013). However, when looking at the term, one does not find a clear unambiguous definition. Often CT is used as a catch-all term for problem-solving strategies (Selby and Woollard, 2013; Curzon and McOwan, 2018). Abstraction, decomposition, generalization, and evaluation around algorithms are techniques of these mentioned strategies. Selby and Woollard (2013) have shown in their paper how multi-layered the term is and that it can be viewed from four perspectives - thinking term, problem-solving term, computer science term, and imitation term (Selby and Woollard, 2013). Despite this multi-layered approach after a search for a definition, no clear explanation was found. In this context, the question arises whether the term will not change in the next few years anyway, since CT is also evolving. In this study, as according to Curzon and McOwan (2018), CT is considered a problem-solving technique, which includes the following important elements: decomposition, pattern recognition, generalization, abstraction, algorithms, and evaluation. Basically, it is about ideas of reasoning and choosing a good representation of data for the problems at hand (Curzon and McOwan, 2018). Furthermore, creativity also plays an important role in the scientific thought process and CT (Curzon and McOwan, 2018; Israel-Fishelson et al., 2021).

Regardless of an imprecise definition of the term, its importance in school is undisputed. CT has already found its way into higher education, but this is not quite the case in primary and secondary education (Settle et al., 2012). In Austria, digital literacy has been a statutory mandatory subject in secondary schools since the 2022/2023 school year, and CT is an important pillar of its curriculum (BMBWF, 2022c). Despite the further development and the integration of CT in various curricula, it is evident that creative and multifaceted problem-solving strategies are indispensable for daily life but also for individual school subjects (Barr and Stephenson, 2011; Selby and Woollard, 2013). Looking at STEAM (science, technology, engineering, arts, and mathematics, or applied mathematics) subjects in secondary school, “Biology” and “Physical Education” may not be perceived as having an obvious connection to CT. However, scientists are showing that the integrated CT concepts in a learning-by-modeling environment facilitate not only a deeper understanding of the subject-specific concepts and processes, especially biological processes (Naulakha and Bar-Joseph, 2011; Hutchins et al., 2020) and physical activity (Fritz, 2022), but may also support developing foundational computing skills and knowledge to support the future learning of advanced computer science (CS) concepts.

There are programs such as “Science through Sports” (Hammrich et al., 2021) but the scientific considerations for the implementation of CT in the sector of physical education are also increasing. This can be done by training analyses but also through problem-solving tasks. A study from Galoyan et al. (2022) combines athletic fields, such as track and field and basketball, with scientific topics, design thinking principles, and CT. In doing so, they also demonstrate many ways in which CT can be implemented through sports into everyday school life (Galoyan et al., 2022).

The same applies to the biological field: During the course “Introduction to Bioinformatic,” biology students were taught concepts and skills related to bioinformatics and CT (such as problem-solving, abstract thinking, and pattern recognition). As a result of the lecture, participants were more engaged in the learning process, and they were able to understand new concepts (biological and CT) (Hong, 2009). As well, Goldberg et al. presented an interdisciplinary course in which CT and computer science concepts were integrated into students’ lectures (e.g., math, biology, and art). To increase engagement and interest in CT concepts and the CS field later, it was tested in areas that might be beneficial and fit the requirements of the biology curriculum (e.g., algorithms for DNA-sequences, or data analysis in health education) (Goldberg et al., 2012). In a more recent study (2020), high school students showed science and computer-assisted learning gains after studying computer modeling within a science unit (Arastoopour Irgens et al., 2020).

### 1.1. The poly-universe game and application In education

**Figure 1:**
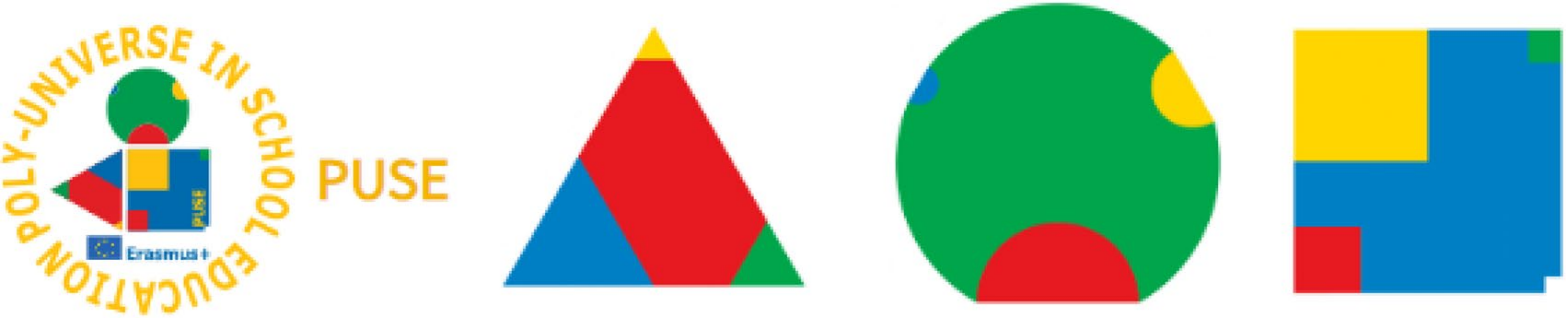
The Project’s Logo (left), and the three Shapes of Poly-Universe Tiles (left) (Saxon, 2018).

“Poly-Universe” (PolyUni) originally was a geometric skill development game, designed by Saxon and Stettner (2019). The game PolyUni consists of 72 unique flat tiles in the form of triangles, squares, and (almost) circles (Figure 1). The novelty of PolyUni lies in the “scale-shifting” symmetry inherent to its geometric forms and color combination system (Saxon, 2018). During the Erasmus+ project called “Poly-Universe in School Education (PUSE),” further work was done on the game and its implementation was researched (Dardai, 2018). The game, originally designed for mathematics classes, is intended to promote geometric understanding and combinatorics, but can further be used in other contexts (e.g., entertainment and non-formal and formal education) (Saxon, 2018; Saxon and Stettner, 2019).

In addition to the analogue game, PolyUni was further developed so that it could also be used as a digital educational tool in primary and secondary education, aiming to enable the application (app) of this game in an online or hybrid learning environment. The online version of the game provides almost all the benefits that the game has in a physical environment, except for direct physical contact with the game sets. The online tasks of this game are enriched with animations based on the rules of the game. Participants can interact with the animations and at the end of the animation, check their knowledge of the game through several quiz questions. Poly-Universe in a digital environment could be also used in the GeoGebra platform (GeoGebra, 2022) for solving problems connected to the game. The methodology of using ICT (information and communications technology) tools during the teaching/learning procedure becomes more and more popular. With GeoGeogebra’s assistance, it is possible to create interactive programs for the creation and further consideration (such as a 3D extension) of the spatial-geometric components that reflect Poly-Universe’s characteristics (Saxon, 2018).

There are no limits to using these individual game elements in the educational field: Besides the game’s initial geometric-oriented tasks like “find the small triangles of the same size and connect them” or “find the small triangles of the same color and connect them” (Saxon, 2018), a variety of creative laying exercises are also practicable and are constantly being developed. During the Erasmus+ PUSE Project in 2008, some studies on the use and the effects of the game PolyUni in the classroom were included (Dardai, 2018). Hoffmann (2020) points out the potential of PolyUni for visualization and sees an opportunity to connect the subjects of “Geography,” “Literature,” “Sociology,” “Political Science,” and “Mathematics” with it. Within the PUSE Methodology and the PUSE study three teaching examples in the subject biology are presented: “substrate and enzyme connection,” “examples of molecules model,” and “characteristics of (flowering) plant families” could be created with the game elements by the student. The “PUSE Methodology” is a material collection of mathematical tasks for primary and secondary school students (suitable for six up to eighteen-year-olds), created within the PUSE project and developed by the project partners (Saxon and Stettner, 2019). The PUSE study was conducted in four different countries (Finland, Spain, Hungary, and Slovakia) to investigate the effects of the game including the following aspects: memory and mental rotation tests, visual perception, attention span, and attitude towards mathematical topics. A variety of methods, like cognitive tests, online questionnaires, and qualitative methods were used for the investigation. The most significant differences before and after testing were found in visual perception, including the isolation and development of shape background ability but also in the students’ attitudes towards mathematics and related tasks. In both areas, the tests showed that the skills and opinions of the students improved significantly throughout PolyUni or changed positively because of the game (Dardai, 2018).

Moreover, besides the implementation in different school subjects, the game PolyUni can also be used within different age groups: from kindergarten (De Vasconcelos Martins et al., 2022) up to secondary school (Saxon, 2018; Saxon and Stettner, 2019). In a 2022 study, the Poly-Universe tiles were used even in Residential Care. The children were in kindergarten and primary school age (3 years and higher). The participants liked the game, because of the colorful elements, simplicity, and universality. The authors even claimed that PolyUni has the possibility to promote social and sensorimotor skills, spatial vision, and algorithmic thinking (De Vasconcelos Martins et al., 2022).

### 1.2. The potential of poly-universe to promote CT

To promote CT, various materials, such as educational games, can be used cross-curricular in different school subjects. Studies and reviews on game-based learning are sometimes contradictory because topic-related research is highly susceptible to a muddle of approaches and methodologies, as to whether games really contribute to learning success or whether they only promote enjoyment in the classroom (Vandercruysse et al., 2012; Hamari and Koivisto, 2015; Crocco et al., 2016; Hamari et al., 2016; Qian and Clark, 2016). A study conducted in 2020 looked at how to develop CT skills through game-based learning: It developed links between CT-based solutions and real-world problems using an adaptive learning game to promote CT skills and CT concepts (Hooshyar et al., 2021). Another option for promoting CT is through the analog educational game Poly-Universe.

In addition to the advantages from a subject-oriented point of view, this game also has an added value in CT. Due to the different variants of combining the parts, different strategies of problem-solving must be applied. “Which parts can I connect to another?,” “If I need a square, what color and shape combinations must be included so that it fits into the figure?,” “If I use this circle now, does that mean that I need a circle in the other color but with the same color as the small circle?” All these questions can happen when students try to create figures with PolyUni (Dardai, 2018). Experimenting with colors, shapes, and sizes in the form of the game offers many possibilities and opens space for creative work around CT and a wide variety of subjects and age groups (Dardai, 2018; Saxon, 2018; Saxon and Stettner, 2019).

Due to the game’s uniqueness and possibility to promote CT als an innovative educational tool (Saxon, 2018; De Vasconcelos Martins et al., 2022), this article aims to show that teachers can utilize the game in other subjects, such as biology (B), digital education (DGE), and physical education (PE). Therefore, three workshop examples, and accompanying teaching and learning materials for B, PE, and DGE were developed by the authors or adapted from existing examples of the “PUSE Methodology. Poly-Universe in school education” by Saxon and Stettner (2019).

### 1.3. COOL informatics – Core concept for poly-universe workshop and material development

The teaching units with the game Poly-Universe were developed as three workshops (W1-3) on the basis of the “COOL Informatics” concept: discovery, individuality, cooperation, and activity. “COOL” as an abbreviation stands for different meanings: firstly for “COoperative Open Learning,” an Austrian teaching model, and second, for “COmputer-supported Open Learning.” Lastly, “COOL” stands for a popular sense of being interesting, motivating, fun, and effective (Sabitzer, 2013; Sabitzer and Stefan Pasterk, 2013).

**Figure 2:**
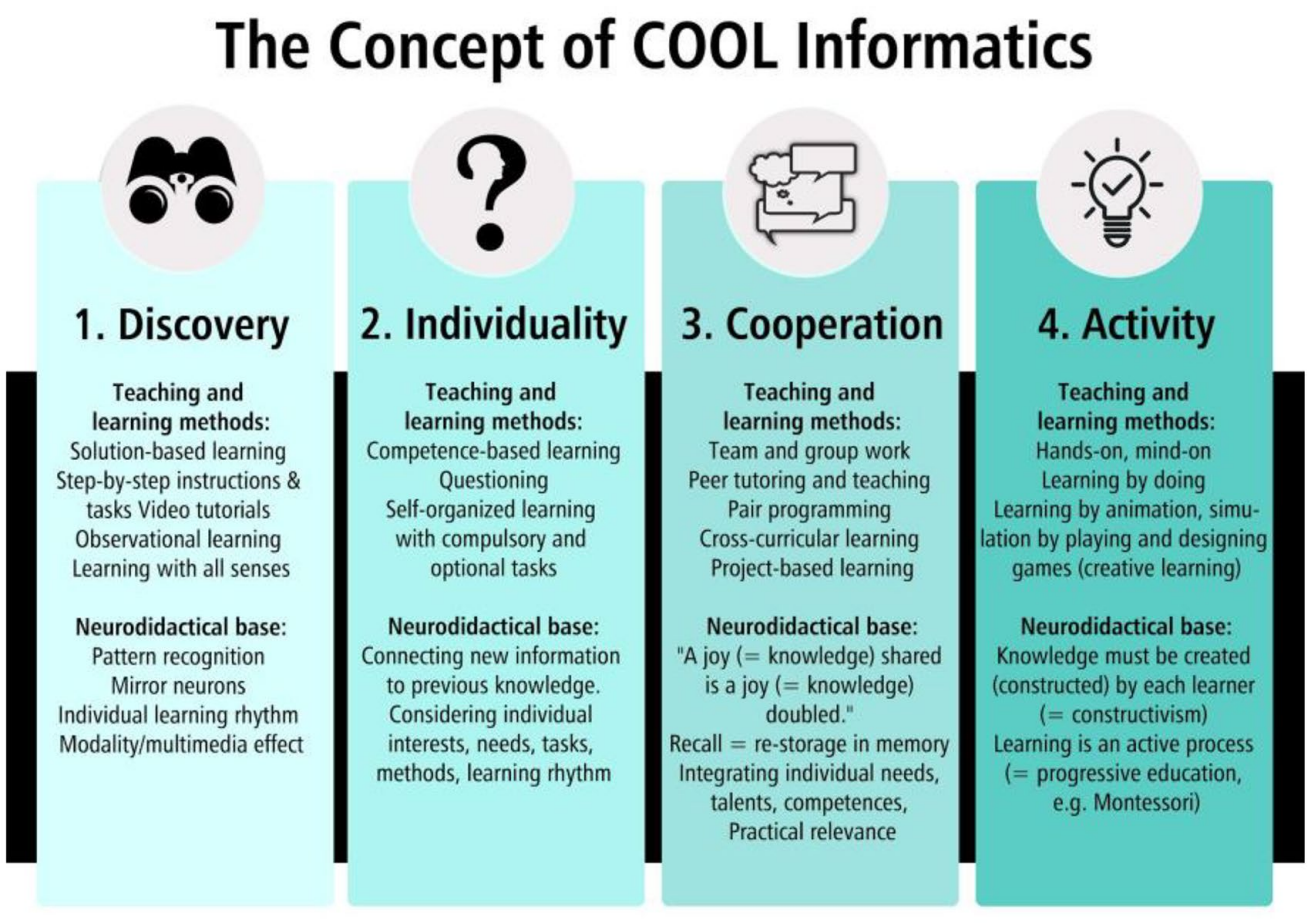
The four pillars of the core concept of COOL Informatics (Sabitzer, 2013).

The concept is a guide for teachers and provides suggestions for innovative, motivating, brain-friendly and supportive teaching that prepares for the demands of the 21st century (21st-century skills) (e.g., communication, collaboration, creativity, and critical thinking skills; Kennedy and Sundberg, 2020). It provides guidance, ideas, and examples for the preparation (planning and material development), the design of teaching units as well as the development and testing of special competencies. Previous research results on the basic concept of COOL Informatics (especially from computer science lessons) show that the consideration of these principles in the design of materials and teaching sequences can strengthen the motivation of learners, contribute to a better understanding of the content, and increase learning success. Figure 2 illustrates the COOL Informatics concept with its four main pillars. Each pillar includes the main teaching and learning methods as well as the neurodidactical foundation, such as pattern recognition, connecting new information to previous knowledge, joy, and constructivism (Piaget, 1971; Sabitzer, 2013; Sabitzer and Stefan Pasterk, 2013).

Within the workshops (W1-3), using step-by-step instructions (e.g., rules of the game), the students can utilize exploratory learning to solve a wide variety of solution-based tasks in their own learning rhythm (discovery). Furthermore, based on competence-based learning, and additional optional tasks with the game PolyUni, the individual needs, strength (e.g., endurance in PE), and interests of the students can be addressed (individuality). With the help of partner work as a teaching and learning methodology, and plenary discussions at the end of the cross-curricular workshops, cooperation, and collaboration within the class are strengthened, joy (together) is conveyed, and knowledge is deepened in the lesson (cooperation). The fourth pillar “activity” is the most important in the “COOL informatics” concept: within hands-on materials, and playing, tickling, and experimenting with the PolyUni tiles, the students are actively learning basic IT concepts and CT skills, by connecting new knowledge with previous one (Sabitzer, 2013; Sabitzer and Stefan Pasterk, 2013).

Moreover, after the task development, planning, and concepting the three workshops, all self-designed tasks, materials (e.g., task sheets, video), and the processes of the workshops were evaluated again or adapted by the research team if it was necessary. After the evaluation, all materials were made available as an open educational resource (OER) to (Austrian) teachers (JKU COOL Lab Materialbörse, 2022; Table 1).

**Table 1:** Overview of all workshops, the CT concepts/skills conveyed in W1-3, and how they were guided by the COOL informatics concept (Sabitzer, 2013; Sabitzer and Stefan Pasterk, 2013).

Workshop	CT-Skills and concepts	Main activities of each phase and how they are guided by COOL informatics
Workshop 1 (W1) Poly-Universe in Biological Education	Abstraction, Generalization, Coding, Decomposition, Pattern Recognition, Algorithmic Thinking, Debugging, Experimenting, Problem-Solving	Explanation Phase: Step-by-Step instruction & tasks descriptions/explanations, observational learning (*discovery*) Developing Phase: learning with all senses, learning by doing, tinkering, experimenting with tiles, learning by playing, and designing creative learning, optional tasks, enjoyment, fun (*discovery, activity, cooperation, and individuality*) Debugging Phase: self-organized learning, group work, competence-based learning (*discovery, activity, individuality, and cooperation*) Evaluation and Feedback: team, group work (*cooperation*)
Workshop 2 (W2) Poly-Universe in Physical Education	Coding, Decomposition, Pattern Recognition, Algorithmic Thinking, Debugging, Experimenting, Problem-Solving	Warm-Up Phase: Step-by-Step instruction & tasks, observational learning, sports activity (*discovery and activity*) Developing Phase: learning with all senses, learning by doing, tinkering, experimenting, learning by playing and designing creative learning, optional tasks, competence-based learning, enjoyment (*discovery, activity, individuality, and cooperation*) Evaluation and Feedback team, group work (*cooperation*)
Workshop 3 (W3) Poly-Universe in Digital Education	Abstraction, Automation, Generalization, Coding, Decomposition, Pattern Recognition, Algorithmic Thinking, Debugging, Experimenting, Problem-Solving	Explanation Phase: Step-by-Step instruction & tasks, observational learning Practice Phase: dance activity, learning with all senses, learning by doing learning (*discovery, activity, individuality, and cooperation*) Developing Phase and Performances: tinkering, experimenting, learning by playing and designing creative learning, project-based learning, fun, enjoyment (*discovery, activity, individuality, and cooperation*) Evaluation and Feedback team, group work (*cooperation*)

## 2. Materials and methods

### 2.1. Research aim

The main goal of this research is to examine creative and innovative possibilities of using the Poly-Universe game in biological, digital, and physical education, to teach simple CS concepts (such as algorithms and coding) and CT skills (such as pattern recognition and abstraction) across disciplines.

For this purpose, three workshops, based on the COOL Informatics concept, were developed. In the first workshop, the Poly-Universe elements were used to map developmental steps of invertebrates in biology lessons (W1). In the second, mathematical tasks were combined with endurance exercises in physical education (W2). Finally, the PolyUni parts were used for dance programming in the third workshop (W3). Therefore, the following research questions are addressed in this article:

1. RQ: How can the Poly-Universe be utilized to promote CT and biological concepts in secondary school biology classes?
2. RQ: How can PolyUni tasks be used to combine physical education with CT in secondary school?
3. RQ: How can the game teach PolyUni CT concepts through dance programming in secondary school?

In addition to the main aim of this work, the following sub-goals were set: Firstly, to investigate how PolyUni can be used to achieve learning goals from the teaching curriculum based on self-designed tasks. Secondly, to evaluate their contribution to the achievement of student learning outcomes, and other possible advantages (e.g., engagement, enjoyment, collaboration).

### 2.2. Data collection and processing

To examine the above-mentioned questions, a mixed-method approach was embodied. The observation of the participants, based on the methodology of the participating observation (DeWalt et al., 1998), was used for qualitative data collection, and an assessment grid and additional picture analysis were used for the quantitative data. The collection process took place in three workshops between June and December 2022. The data was processed *via* an evaluation of a self-designed computational thinking curriculum assessment grid (CTC-AG).

In regards to the participating observation method (DeWalt et al., 1998), the participants’ options regarding the entertainment factor of the game, the misunderstanding of the tasks, the collaboration with peers, and the problem-solving process, were observed and documented by the research team. The participant observation is a method, which has its origins in the field research of De Gérando (2016), was carried out at secondary schools, in a natural environment for the students. Participatory observation was chosen as the data collection method, in order to survey the participating students in natural way whilst they were using the game (in terms of fun, enjoyment of the tasks, behavior within class), and while solving the tasks (understanding the question and rules of the game, correctness of the tasks, errors, debugging). Therefore, this approach collected qualitative data that was very useful in allowing the research team to receive a clearer picture of how the students were using the tiles of the PolyUni game, and additionally, how the participants were interacting with each other. Further, the researchers were openly recognizable to all students and actively participated in the lesson for, e.g., taking pictures during the lectures, explaining the game PolyUni and tasks, and assisting throughout the exercises. The research team took notes during their observation process. In addition, data was documented *via* photographs throughout all workshops, taken by the students themselves in W1 and W3, and the research team in W1-3. At the end of W1, all photos taken during the lectures were sent to the workshop leader for evaluation and analysis.

The CTC-AG was answered manually by all members of the research team for each workshop, partly during and the rest after the lecture, using the notes from the observation (e.g., written recording, notes) and pictures. The questions regarding the game and task design, collaboration within the group, and enjoyment could be answered due to the observation and this documentation process during or shortly after the workshops. The rest of the questions, regarding the accuracy of performing CT tasks, biology practices, the dance, or sports exercises, and further the achievement of the pre-defined teaching and learning goals in all subjects, built on the Austrian secondary school curriculum (BMBWF, 2022a) which is based on bloom taxonomy (Bloom et al., 1956), were processed and checked *via* the CTC-AG after analyzing the pictures and the task sheets (only in W2).

The evaluation criteria of the CTC-AG are based on the Austrian school law (BMBWF, 2022a) and the AHS (German: “Allgemeinbildende höhere Schulen”; General secondary school) curriculum for secondary schools for the subjects of digital literacy, biology, and physical education by the ministry (BMBWF, 2022b). The CTC-AG contains on the one hand of general and demographic questions regarding the number of participants and groups, the location and date of the workshops, age, and gender of the students, and on the other hand of 21 questions that are summarized into three main topics: game and task Design (“Task”), the curriculum biology, physical education or digital Education (“Curriculum”), and “CT” (shown in the Appendix).

The questions can be answered on a scale from “I-V” Likert-5-Scale (Likert, 1932). The degree of correctness or fulfillment of a questionnaire item was rated according to the Austrian school grading system from “I” to “V” (1–5) by the scientific team. “I” stands for “the question has been answered far beyond the essentials,” “II” for “beyond the essentials,” “III” for “fully met in the essential areas,” “IV” for “largely fulfilled in the essential areas” and “V” for “not fulfilled in the essential areas” (BMBWF, 2022a).

### 2.3. Sampling of the workshops (W1-3)

To analyze and evaluate the utilization of the game PolyUni in secondary school, three different PolyUni-workshops were held between June and December 2022 an Austrian secondary school (BRG Steyr/Upper Austria): “Poly-Universe in Biological Education” (W1), “Poly-Universe in Physical Education” (W2) and “Poly-Universe in Digital Education” (W3). All of the participants had no previous knowledge and did not know the game PolyUni until the day of the workshop.

**Figure 3:**
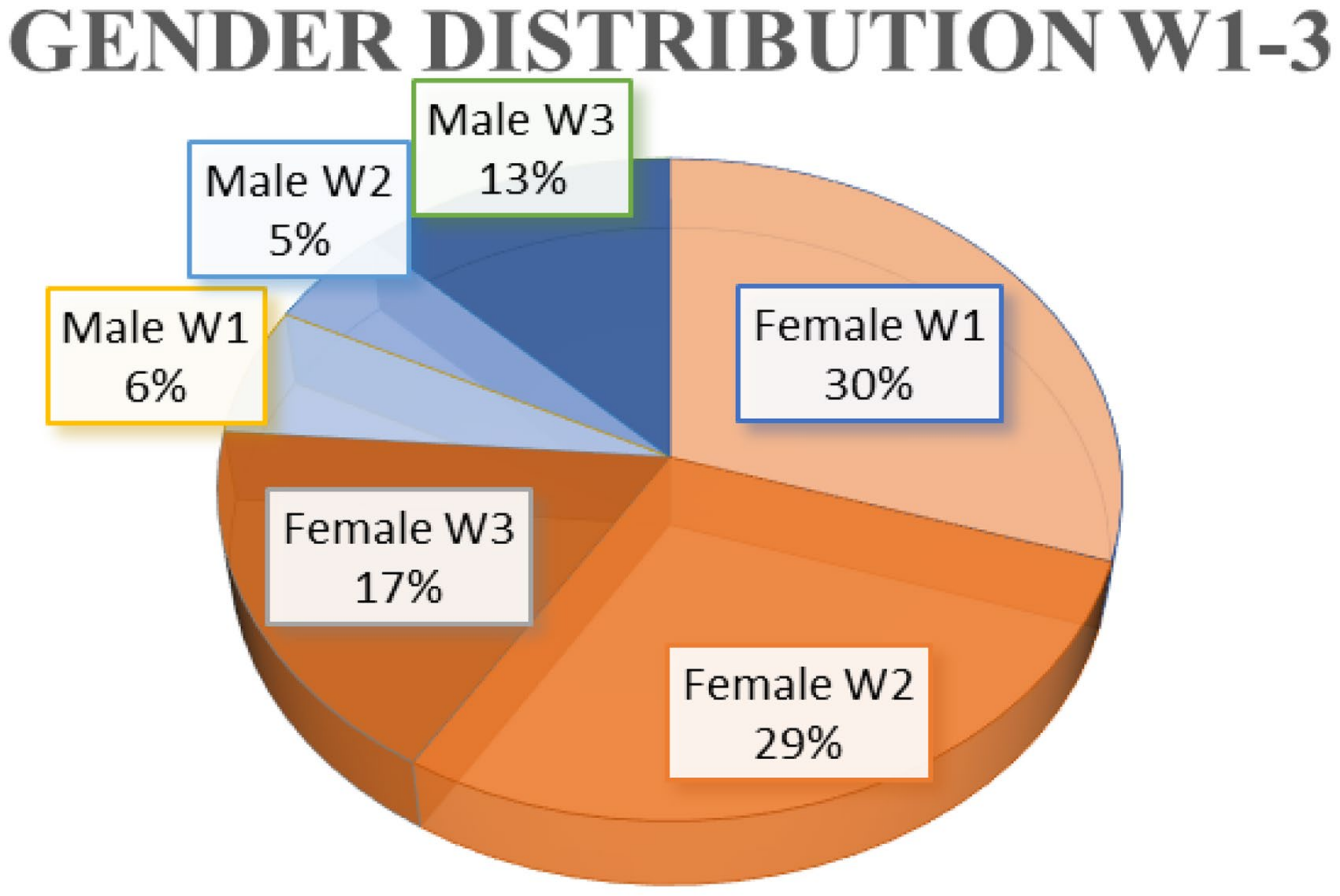
Gender Distribution of the participants in all held workshops.

In sum, 80 11–12 year–old Austrian students participated in this research, 69 identified themselves as female, 19 as male. Each of the three workshops lasted 100 min, and two researchers were always present per lecture. On 15th June, 2022, twenty-nine 11–12-year-old students (female = 24, male = 5) attended the first biology workshop (W1). On 30th June 2022, the second workshop for physical education took place, and twenty-seven students (female = 23, male = 4) participated (W2). Lastly, the third workshop took place on the 2nd December, 2022. In this lesson, twenty-four 11–12-year-old students (female = 14, male = 10) participated in the dance programming workshop for digital education (W3) (Figure 3).

### 2.4. Task and course design: W1 “poly-universe in biological education”

The first workshop “Poly-Universe in Biology Education” was intended for the subject Biology, but can also be taught interdisciplinary in the subject DGE. In W1, information about invertebrates was conveyed and discussed. Some of the biological topics were already known by the students (such as the morphology of insects) and some were not taught before (such as the morphology of arachnids and the life cycle of insects). The topics were given to the students as worksheets to supplement their textbooks. The different tasks and the worksheet were, based on the Austrian biology AHS curriculum, self-designed, and “Bio@school 2” (Weisl and Schermaier, 2021) was used as the textbook, because “Bio@school 2” is the biology textbook at this Austrian school and all students have this available. The students should use the information to solve the following three tasks, using the PolyUni game: Task one: What is the morphology of a spider compared to an insect? Task two: What is the life cycle of a cockchafer? Task three: What are the differences and similarities between bugs and beetles? The desired teaching and learning objectives for each subject, biology, and digital literacy were recorded based on the Austrian Curriculum (BMBWF, 2022b) in advance so that the teaching unit can then be evaluated using the CTC-AG.

The following learning and teaching goals have been defined: The students recognize, describe, and distinguish the structure and shape of insects and arachnids. They can recognize, name, and describe the differences and similarities between bugs and beetles, and the development cycle of a beetle. The students discover similarities, rules, and patterns in PolyUni. The students use and create codes with the game, to help them name, understand and describe biological processes from the everyday life of an insect and a spider. The students follow the game’s clear instructions and carry them out. Furthermore, the students can recognize and correct mistakes in their and other codes themselves.

In addition to these goals, the course of the workshop was also determined in advance, and its duration (100 min) was set as follows: In the first phase (10-20 min), the Erasmus+ project PUSE, the worksheet, the game, and the tasks were presented in the Explanation Phase and the students were divided into five groups. The groups were separated by drawing lots of the different PolyUni game pieces. Each group received a set of the PolyUni tiles, wooden sticks, blank cards, pencils, and a worksheet with the same three tasks. To solve the tasks, the students had to use the game PolyUni, the worksheet, and the textbook. Pens, cards, and wooden sticks were optional. In the second Development Phase (50-60 min) the students solved the tasks with the game and took a photo for documentation afterwards. The first two tasks had to be completed by the students in 100 min, the third task was optional. After the completion of each task, the group members looked at the results of the other groups, to find any mistakes, and analyzed different approaches, color, shape, or size codes used. Thus, they could revise, change, or even contribute to their solutions to start their solving process. After this Debugging Phase (10 min) the students took a second photo. In the final Evaluation and Feedback Phase (10–20 min) the (biological) content was repeated and the students’ results were discussed.

**Figure 4:**
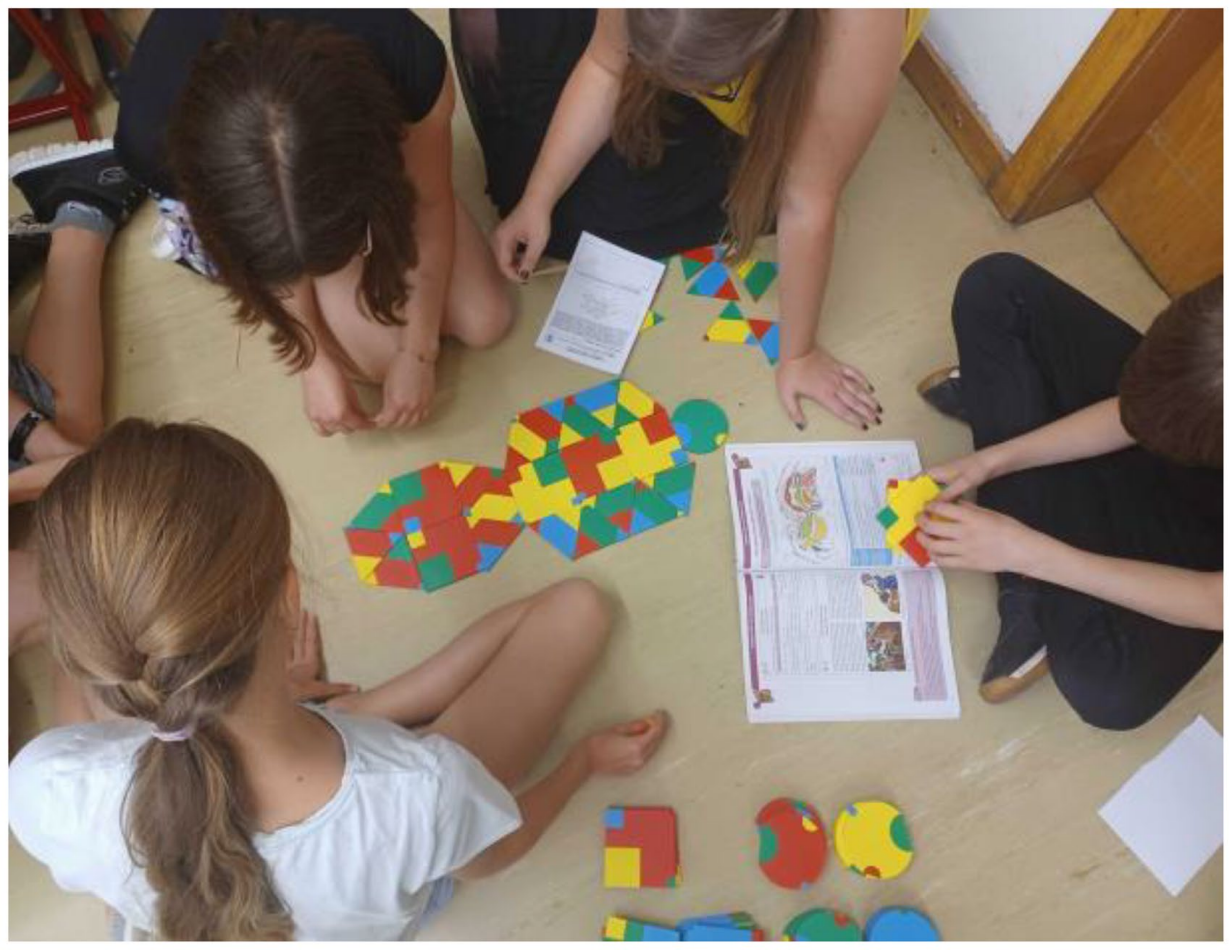
Students solving biology tasks with PolyUni files in W1.

The workshop at the secondary school proceeded as planned: Explanation Phase (20 min), Development Phase (60 min), Debugging Phase (5 min), and Evaluation and Feedback Phase (15 min). The only deviation took place within the Debugging Phase, which had been integrated into the Developing Phase. Only one of the 5 groups took a photo after their first attempt to solve the task, then observed the results of the other groups, and afterwards discussed and modified their results, and documented their final approaches within a second picture. All remaining groups of students wandered through the room during the entire processing time of the Development Phase, observed the other solutions, and thus constantly changed their approaches without visually recording the individual steps, but only the final results (Figure 4).

### 2.5. Task and course design: W2 “poly-universe in physical education”

The second workshop “Poly-Universe in Physical Education” was developed for the subject PE and interdisciplinary for the DGE. The main focus of W2 was on the potential of physical activity to enhance students’ cognitive functions by improving their CT skills. In addition, the students should be enabled to expand their motor skills in a variety of ways and to further develop their conditional skills. The lesson was also intended to improve the students’ coordination abilities and to raise their awareness of their own movement behavior in terms of movement quality and movement economy. To connect all these predefined teaching goals, an alternating task model was used in which cognitive and physical tasks rotated with each other according to a predefined time window.

**Figure 5:**
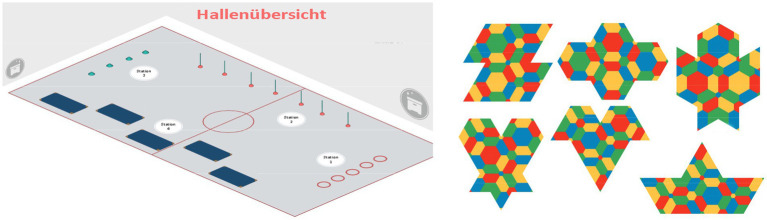
Overview (German: “Hallenübersicht”) regarding the obstacle course and “Tangram” tasks in W2 (Saxon and Stettner, 2019).

For the physical task area, an obstacle course (as shown in Figure 5) with four different movement challenges was chosen. Each challenge contained a physical component (basic movements such as jumping, running, and/or a combination) and a cognitive part (e.g., movement reaction to certain colors). In relation to the cognitive section of the lesson, the task “Tagram” from the script “PUSE Methodology Poly-Universe in school education” by Saxon and Stettner (2019) was used. “Tagram” is about reconstructing given shapes with connections of the same size and color by using the PolyUni triangle set.

The lesson had a total duration of 100 min and was divided into a Warm-up phase (20–25 min), a Development phase (25-30 min), and a final Evaluation and Feedback phase in the plenum (10-15 min). Transitional phases for the assembly and disassembly of equipment were calculated with a duration of ten to 15 min each.

#### 2.5.1. Course of W2 “poly-universe in physical education”

In the beginning, the students were informed about the goals and contents of the lesson. Then, to generally warm up the students, the movement game “Treasure hunt” was introduced. After the Warm-up Phase was over, the students were gathered into six groups. After group selection, the next section of the lesson “Tangram” was explained (Saxon and Stettner, 2019). Therefore, the previously created teams were given organization cards to introduce the subsequent set-up phase. Together, an obstacle course was set up, the extent of which was oriented along a volleyball court. Within the volleyball court, each team was given a small workspace at a distance of about two meters from the other team’s workspaces as well as from the obstacle course. Finally, one triangle set of the game was placed on each of the individual workspaces. Before the game began, the students were asked to sit in pairs in their assigned workspace. The teacher then explained the features and rules of the game “Tangram.” The goal was for the students to work together to build a given geometric figure using the PolyUni tiles. The level of difficulty could be adapted to the students’ performance and experience.

In addition to the “Tangram”-puzzle (Saxon and Stettner, 2019), team members took turns completing the previously constructed obstacle course. The number of laps to be completed was depending on the performance level of the students (e.g., two or three rounds). Before the start, each team received a small card on which the figures to be created were shown. If a team succeeded in solving a figure, another shape had to be solved. After a predefined period (20-25 min) the game ended with an agreed signal. The winner was the team that was able to solve the most puzzles together. At the end of the game, all materials and equipment were put away by the previously assigned students. Finally, there was a reflective discussion in the plenary, where the students were given the opportunity for feedback and open questions about the lesson. The teacher also referred again to the main points of the lesson mentioned at the beginning and asked the students content-related questions.

### 2.6. Task and course design: W3 “poly-universe in digital education”

The workshop “Poly-Universe in Digital Education” was about algorithmic dancing using codes, mapped with individual PolyUni elements. Application examples of this workshop content are in addition to the subject DGE, interdisciplinary also the subject PE, or computer science (CS). Within this teaching unit, students learned playfully and dance-wise with algorithms. Students could clearly name and describe instructions for action and execution. Aim of W3 was that all students learn the concept of programming using simple dance movements. After the learning unit, the participants knew how to dance the shown movements and could guide them using the PolyUni elements. At the end of two teaching units, all students mastered the movements of the PolyUni dance programming and their own choreography. Furthermore, they could create, read, and understand PolyUni codes as dance instructions, and could thus apply simple CS concepts such as algorithms.

**Figure 6:**
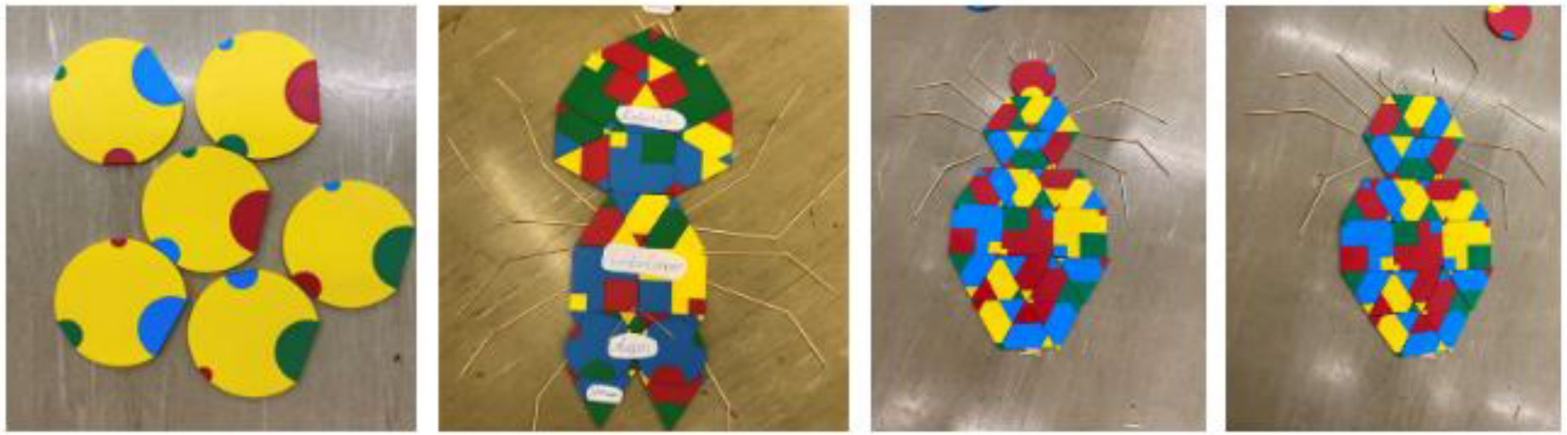
Students’ solutions to represent the eggs of a cockchafer (left), spider morphology (middle left), and the first (middle right) and final (right) approach of a spider morphology.

The two teaching units were divided into the following phases (Figure 6): “Explanatory Phase” (10–20 min), “Practice Phase” (30–40), “Development Phase” (20–30 min), “Performances” (20 min), “Evaluation and Feedback” (5–10 min). In the “Explanatory Phase,” the game and its rules, the worksheet with the dance (see Figure 6) and algorithms in general were explained. In the “Practice Phase,” the students first practice the individual movements within groups. At the end, the given dances were danced in the plenum as a class. In the “Development Phase,” new code and dances were created within six small groups, which were presented to the class in the “Performances” phase. At the end of the workshop, in the “Evaluation and Feedback,” students’ opinions on the game and regarding the tasks, and their fun-factor are evaluated and written down, using the grid. During W3, the research team took pictures and notes for the final evaluation grid.

#### 2.6.1. Course of W3 “poly-universe in digital education”

The individual phases went as planned. The explanatory phase and the introduction to the topic “algorithms” took longer (25 min), but the students could already dance the codes shown much faster than planned (20 min), and read the dance introduction in the plenum. Furthermore, they were able to gather in small groups in the first hour to develop their own dances and codes with the Poly-Universe parts. The second session ran as prepared in advance.

## 3. Results

**Figure 7:**
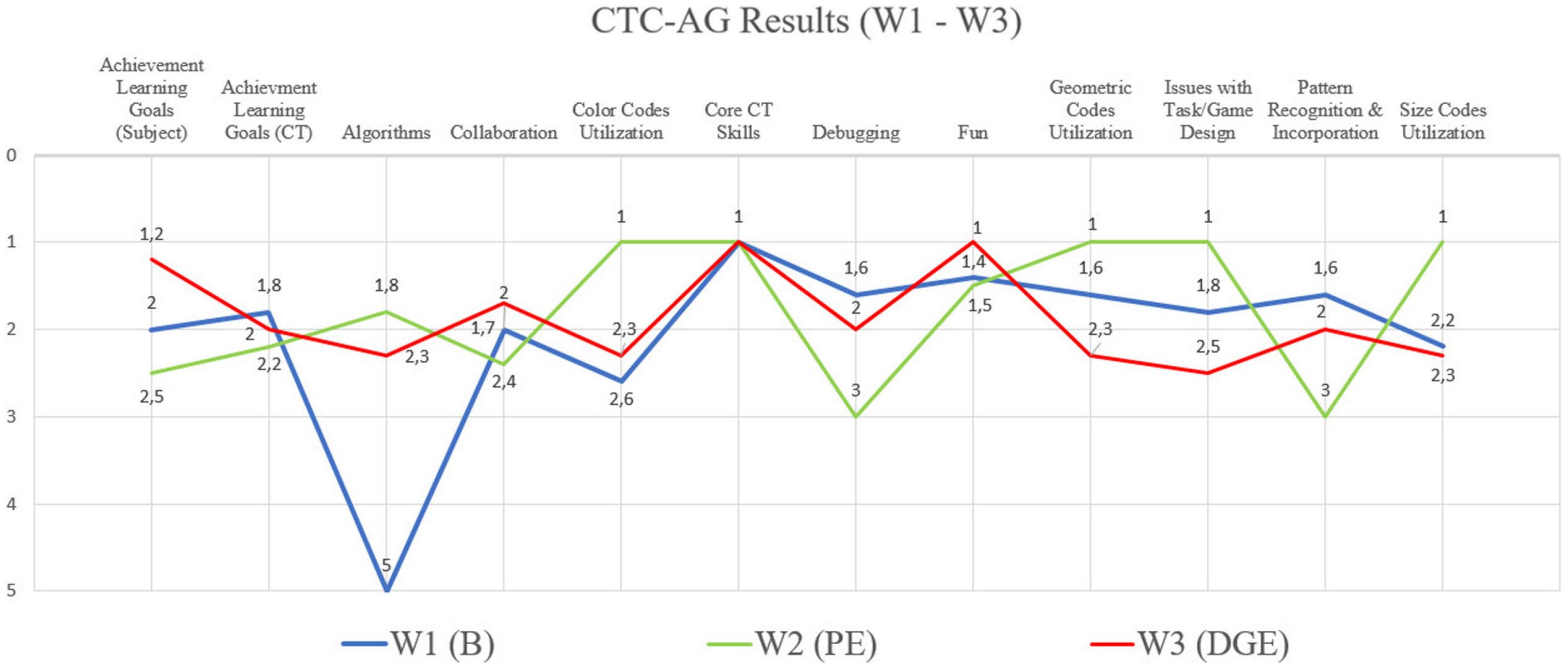
Results of the comparison of all participating groups from workshops 1, 2 and 3. The Y-axis shows the grades 1–5, according to the researcher, and the X-axis is showing excerpts of the CTC-AG items.

Figure 7 shows the comparison of the observers’ opinions of all participating groups from all three workshops (W1–3). The results of the individual groups were evaluated using the Austrian school grading system (1–5) and in the table, the average value (arithmetic mean) of the findings is given. All authors manually filled in the grid and the average value of the results of all answers was taken as the final result. The concordance relating to the comparison of the individual workshops among the research team according to filling out the CTC-AG for each workshop was 89%. These data indicate that the results obtained are valid. A complete table containing all groups and answers according to the research team, is in the Appendix (There, the groups are described as numbers from “1 to 6″ in the corresponding row and column: “1″ stands for group number 1, “2” for group number 2, and so on. Further, a list with all 21 questions (Q1–21) of the CTC-AG is also listed).

In the first category “Task” of the grid regarding the task and game design, all groups achieved an average score (school grade) of 1.7 in W1, 1.1 in W2, and 2.1 in W3. Thus, in the second workshop, there were the fewest issues in terms of task understanding, and explanations of the game or the exercises, especially in W3, shown in Figure 7 (“Issues with task/Game Design”). Overall, there were hardly any problems with task understanding in all W1-2.

Regarding the curriculum, the predefined teaching/learning objectives in the subjects B, Pe, DGE were also achieved above average in all workshops, especially in W1 (1.2). Concerning the achievement of the learning goals regarding CT, all participating groups of students achieved near-top grades.

Looking at the usage of the game PolyUni in relation to “Core CT Skills,” according to the research team, the goals are achieved beyond measure in all workshops (1): all students used CT skills (such as problem-solving, pattern recognition, dividing problems into sub-problems, tinkering) in order to solve complex subject-specific problems using the tiles (1.0). In addition, all the children were able to present the illustrations of the animals in a simplified manner (abstraction) in order to generally represent the most important characteristics of, for example, insects and spiders (generalization). Furthermore, they had to In addition, strikingly, students did not use algorithms in W1, but very well in W2 (1.8) and W3 (2.3).

In summary, according to the research team, it can be seen that in all workshops the previously defined teaching/learning goals were achieved, and that the students also had a lot of fun in the lessons (W1 = 1.4; W2 = 1.5; W3 = 1), and worked well together in peers to solve the tasks, especially in W2 (1.7). In the following, the results of the individual workshops are described in more detail and illustrated with additional image material.

### 3.1. Results: W1 “poly-universe in biological education”

**Figure 8:**
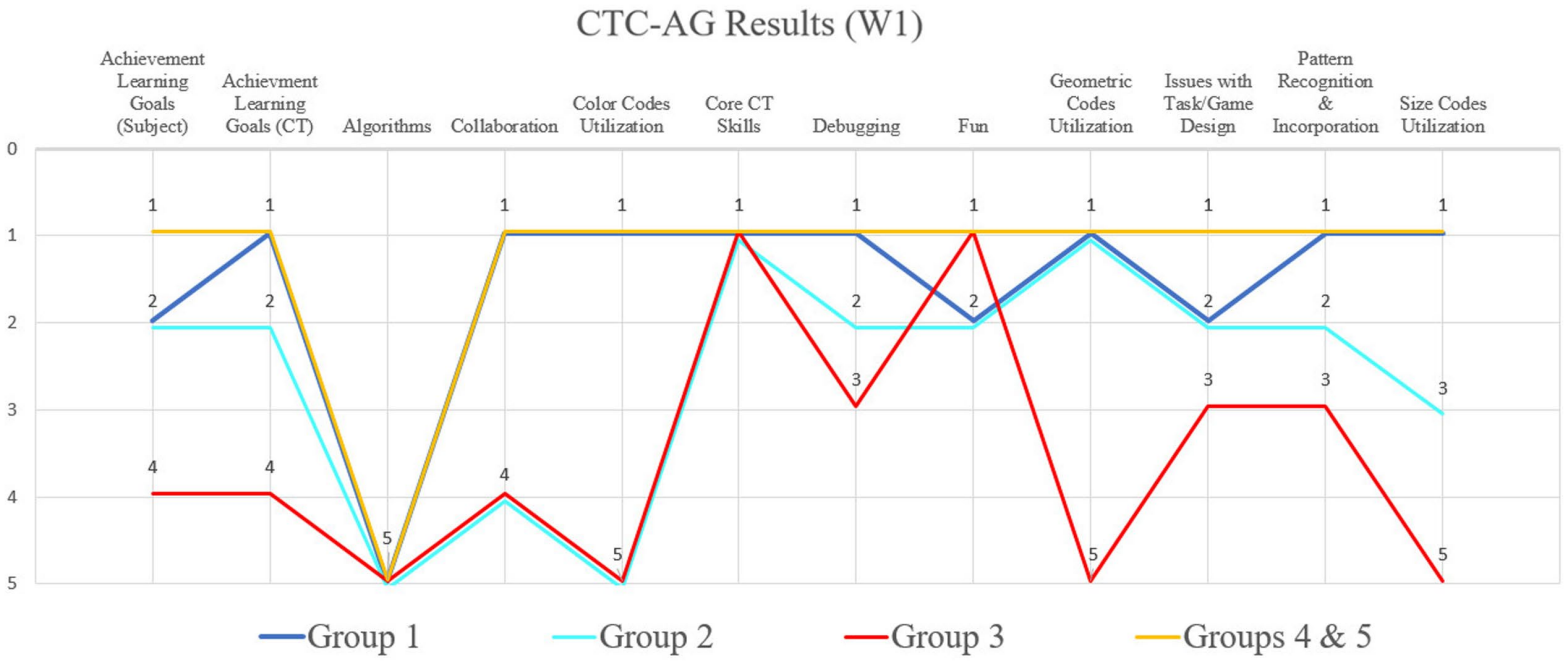
Results of the comparison of all five participating groups from workshop 1. The Y-axis shows the grades 1–5, according to the researcher, and the X-axis is showing excerpts of the CTC-AG items.

**Figure 9:**
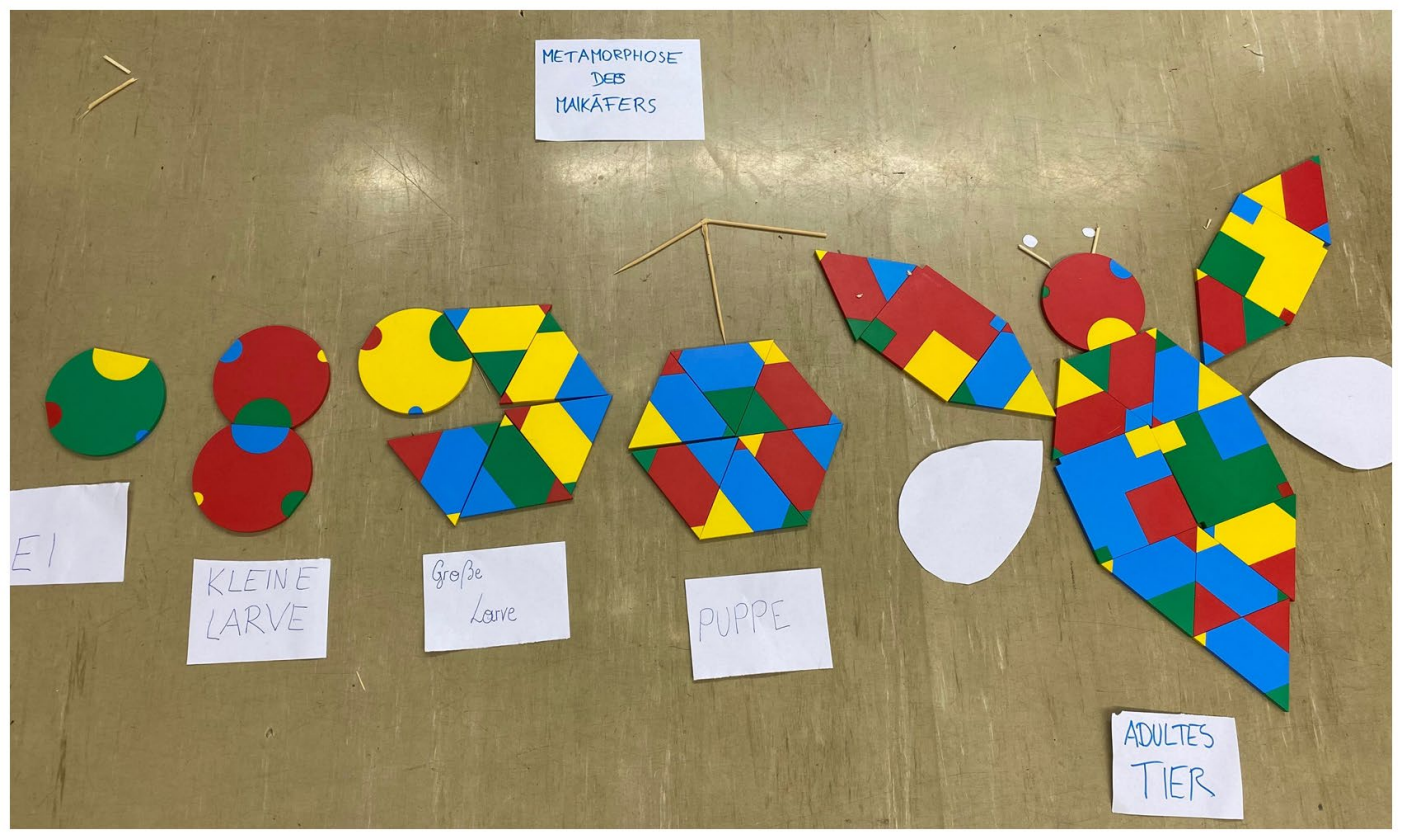
Solution of task 2, representing the life cycle of a cockchafer.

Regarding the task and game design in W1, all groups understood the rules of the game and the tasks “fully met in the essential areas.” Furthermore, all groups could start solving the tasks with only a little help from the research team. Group 3 needed the most help and groups 4 and 5 the least (Figure 8). It is significant that all five groups enjoyed the use of PolyUni in biology lessons “far beyond the essentials” or “beyond the essentials.” This result indicates that the initial problems with the task and the explanation of the game had no impact on the fun in the workshop, and, furthermore, PolyUni creates enjoyment in biology class. Concerning the results of tasks in reference to the Austrian biology curriculum, the findings show that groups 4 and 5 could solve the tasks perfectly. Figure 9 shows group 4’s solution to task 2, presenting the life cycle of a cockchafer.

Using the tiles of the game, the students were able to represent the different stages (egg, larva (younger and older), pupa, adult animal) in a biologically correct manner and label them correctly using the cards. Groups 4 and 5 had no problems in understanding and presenting previous and new learning content. Two groups (1, 2) were partly able to understand and present previously learned and new biological content with the game. It is evident that group 3 struggled the most in biological correctly solving the tasks. After comparing the solutions of the individual groups, it turned out that 4 out of 5 groups had achieved the specified teaching and learning goals in biology, but group 3 only “largely fulfilled” the tasks “in the essential areas.” In contrast, groups 1, 2, 4, and 5 achieved the teaching and learning goals set in advance “far beyond the essentials” or at least “beyond the essentials.”

These results indicate that PolyUni can be successfully used to reach the required teaching and learning goals in biology education. Regarding CT, all students were tinkering, playing, and experimenting with different tiles of PolyUni to solve the tasks. All groups could break down a complex biological problem into sub-problems and thus solve the required tasks. Furthermore, all students used PolyUni to identify similarities, differences, and patterns to solve complex biological problems. In addition, all five groups could hide unimportant details so that the participating students could focus on the essential aspects of the problem and thus solve the task.

After photo analysis, the findings show that groups 1, 4, and 5 used color, size, and (geometric) shape combinations as codes to correctly display the biological content and to solve the different tasks. In Figure 6 groups 1 and 5’s solutions are presented. Group 1 used the same sizes, the same-sized semicircles pointing in the same direction, same shapes (circles) and color (yellow) to represent the eggs of a cockchafer. Same size, color, and shape codes are also used in group 5’s solution. Two green triangles of equal size are used for the spider’s jaw claws and circles for the eyes. As seen on the prosoma two of the same size semi-squares are connected vertically with each other. Group 2 used shape combinations as codes “far beyond the essentials” and the size combination utilization was “fully met in the essential areas.” Only group number 3 hardly used geometric shape codes, and furthermore, no color and size codes. Concerning the debugging, at least all groups “fully met in the essential areas.” From the observer’s point of view, all students found mistakes and improved them on their own, recognized patterns and codes from the other groups, and further incorporated them into their own codes to improve their solutions, especially in groups 1, 4, and 5. As seen in Figure 6, group 4’s first approach was that Araneae have a caput, a thorax, and an abdomen, i.e., a body structure like an insect. After comparison with the other groups, their first approach was improved, and the final solution showed the prosoma and the opisthosoma.

After analyzing the photos, the findings indicate that with the utilization of the game the previously defined teaching and learning goals for CT were fulfilled “far beyond the essentials” for groups 1, 4 and 5, “beyond the essentials” for groups 2 and 3 only “largely fulfilled” the tasks “in the essential areas.” These results regarding the learning and teaching goals in CT and biology appear verified after comparing the intensity of collaboration within the different groups: The students of groups 1, 4 and 5 collaborated in peers “far beyond the essentials” to solve all problems and tasks together, but groups 2 and 3 only “fully met in the essential areas” in this category. In summary, with the results concerning CT, it can be assumed that the game PolyUni can be successfully used in biology classes to promote CT in digital literacy in secondary school.

### 3.2. Results: W2 “poly-universe in physical education”

**Figure 10:**
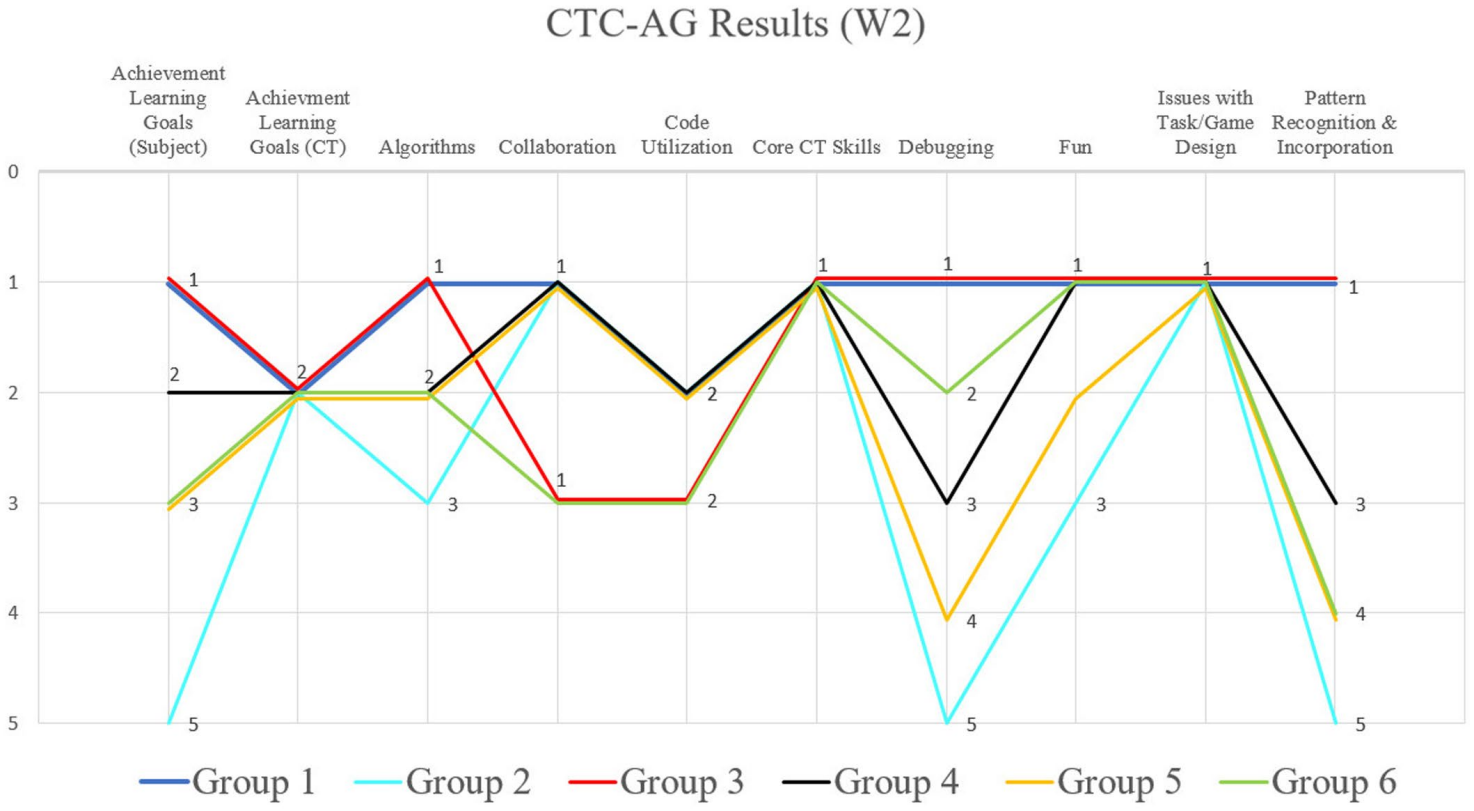
Results of the comparison of all six participating groups from workshop 2. The Y-axis shows the grades 1–5, according to the researcher, and the X-axis is showing excerpts of the CTC-AG items.

Results related to the game and task design suggest that all groups understood both the task and the function of the game after the teacher explained it. The same results could be found regarding task comprehension. Furthermore, it was shown that all six groups were able to initiate the game without additional explanatory aids after the teacher’s introduction. It also became apparent that the game was particularly enjoyable “far beyond the essentials” for most of the participant groups (1, 3, 4, and 6). As for the results concerning the Austrian physical education curriculum, the data should be interpreted with caution. Since PolyUni was embedded in the lesson structure only as a cognitive subsection, student experiences with similar tasks were included in the analysis. With this information in mind, the data provide preliminary evidence to suggest that with the help of PolyUni, most of the students may appropriately apply and convey prior information regarding previous contents of PE lessons. Considering that the task “Tangram” (Saxon and Stettner, 2019) was already the content of the physical education of all group participants in its original variation, it was unsurprising that the game allowed the students to accurately define and explain prior knowledge. Minor difficulties were encountered in the transition from the original game idea “Tangram” to the variant with the game’s elements. Only students from groups 1 and 3 were fully capable of accurately comprehending and presenting. and explaining new material, whereas the remaining groups needed additional explanations in the introductory part of the lessons (Figure 10).

A similar picture emerged during the learning goal orientation, whereby group 2 was the only group that could not fulfill the predefined teaching goals from the observers’ point of view. At the same time, results regarding CT yielded some interesting findings. First, the data showed that all students were able “far beyond the essentials” to complete the objective by exploring, playing, and/or tinkering. Additionally, every group seemed to be able to divide a difficult problem into smaller ones and complete the assignment. To address challenging issues, all students also seemed to utilize PolyUni to find patterns, similarities, and contrasts. Moreover, all students were able to find patterns, similarities, and contrasts to tackle complex problems. Differences between the individual groups only became clear in the way the tasks were processed. It was found that groups 1 and 3, and to a lesser extent group 4, were particularly good at blocking out unnecessary details and avoiding attentional distractions to solve the task. Group 5 and 6, on the other hand, managed the task with some difficulty regarding task focus, whereas group 2 frequently excelled due to distractions from various group members. Furthermore, possibly due to the basic idea of the game “Tangram,” the data showed that all groups were able to adequately use geometric figures as well as color and size combinations to accomplish tasks.

**Figure 11:**
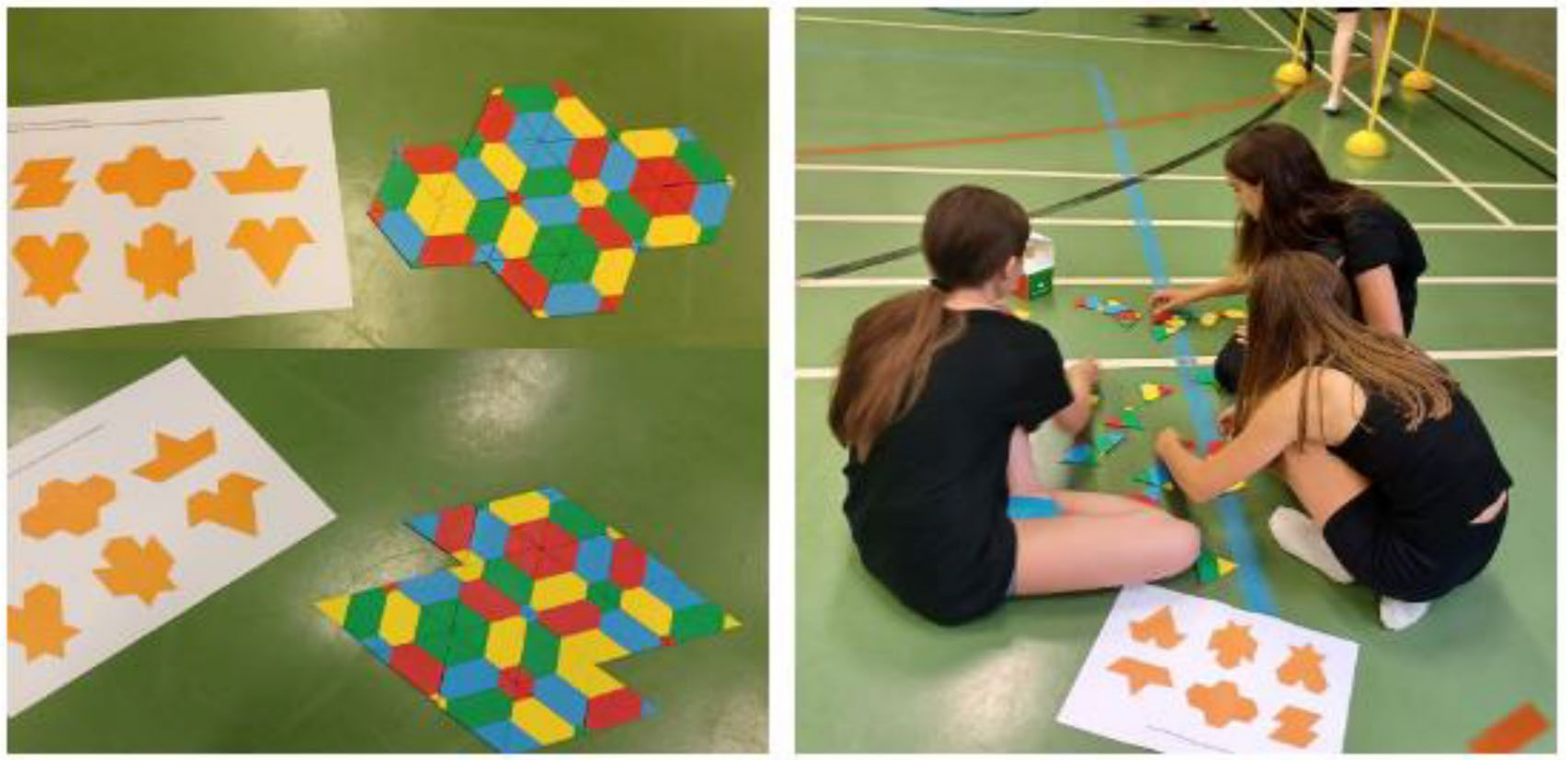
Solutions of “Tangram” (Saxon and Stettner, 2019) tasks in W2.

However, major differences were revealed in the detection and processing of faulty work steps. Whereas groups 1 and 3 identified and corrected errors independently, the remaining groups sought help from the teacher to varying degrees to solve the task. A similar trend was again reflected in the ability to use algorithms in a task-specific manner. This showed that groups 1 and 3 stood out particularly positively, while group 2 required assistance more frequently. However, commonalities emerged in the collaboration between individual group members of each team, as a high proportion of teamwork was observed in all groups. Finally, it became apparent that by using PolyUni, the teacher was able to complete the teaching goals and learning objectives for CT and digital literacy to a full extent in groups 1 and 3. In groups 4, 5 and 6, the task only partially met the predefined learning objectives and for group 2, the task was found to be unsuitable from the observers’ point of view (Figure 11).

### 3.3. Results: W3 “poly-universe in digital education”

**Figure 12:**
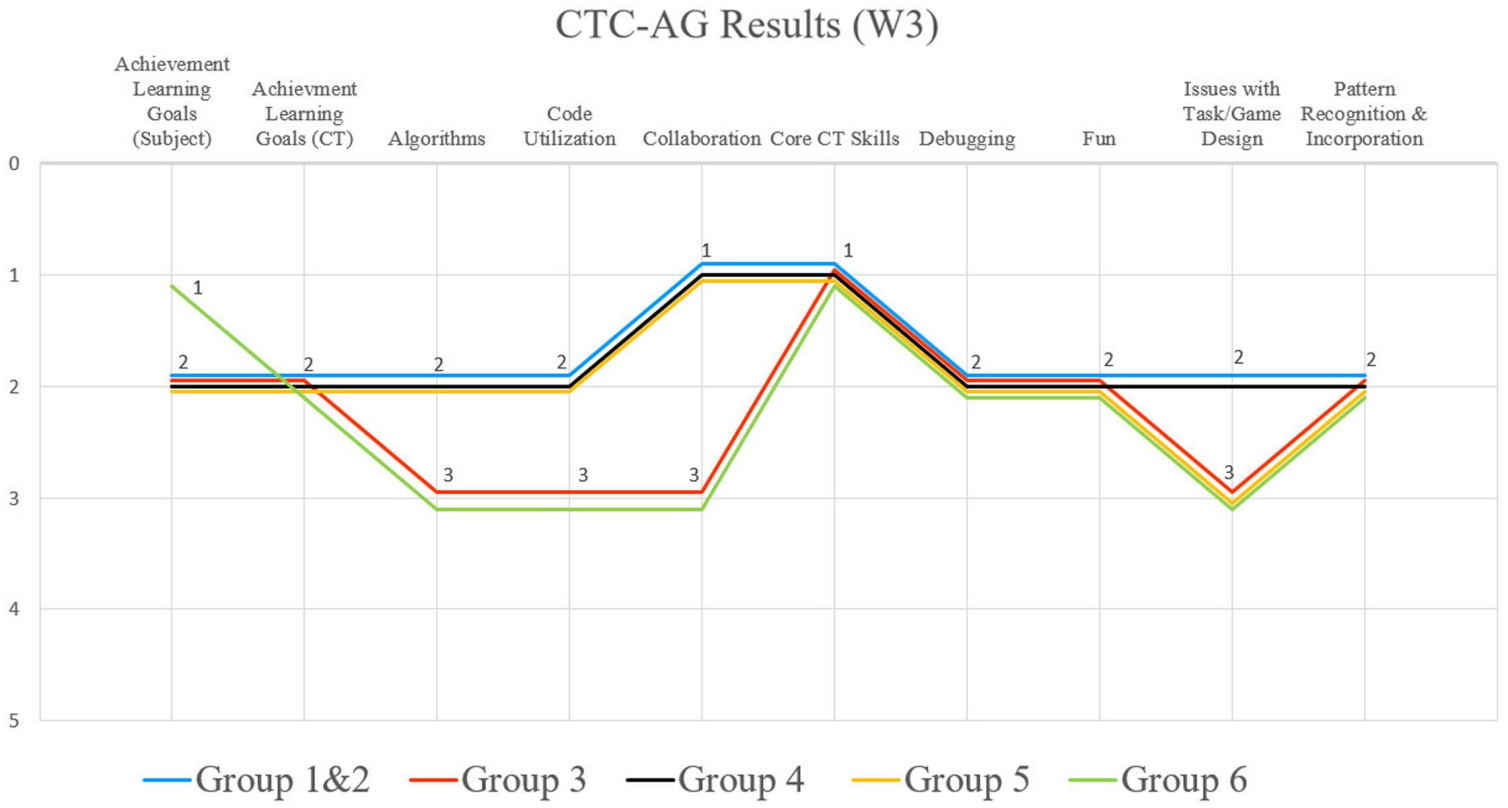
Results of the comparison of all six participating groups from workshop 3. The Y-axis shows the grades 1–5, according to the researcher, and the X-axis is showing excerpts of the CTC-AG items.

In W3, all students of the six groups at least “fully met the essential areas” by understanding the tasks and the rules of the game. All groups only needed a little support by the research team but had a lot of fun with the dance programming (Figure 12).

**Figure 13:**
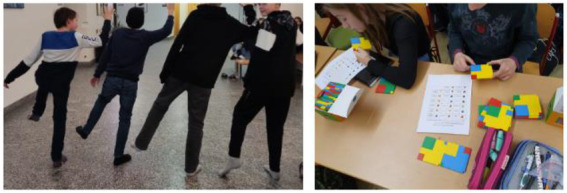
Students in W3 are dancing their own dance programming (left) and creating new codes for every movement using the PolyUni tiles (right).

With regard to the “Digital Education” curriculum, all students also achieved at least “beyond the essentials” the previously defined teaching objectives. Neither in the category “Task” nor “DGE Curriculum” did one group stand out particularly prominently in one direction or the other. All participating children of W3 were on a very similar level and there were hardly any differences. This is also evident in the “CT” category. All groups played and experimented with the PolyUni parts and created new codes for individual dance moves. Particularly many and different codes for dance movements (between 5 and 13 different codes; group1: *f* = 13) were created by groups 1, 2, 4 and 5, but over all, all participants developed new movement sequences, as shown in Figure 13.

After the photo analysis, it is striking that all groups found mistakes on their own (de-bugging) and could improve their dance algorithms on their own, without any additional help from the research team. Furthermore, all participating students could recognize patterns and (dance) codes with the PolyUni tiles from other groups, and incorporate them into their own codes and representations to improve their final approach. Therefore, all predefined objectives were achieved at least “beyond the essentials” with regard to CT.

## 4. Discussion

Even if at first glance the connection between CT and such diverse subjects, especially B and PE, seems impossible, previous studies show that this is not (Hammrich et al., 2021; Galoyan et al., 2022). However, in order to be able to make general statements more scientific research must be done in this field in the future. The game Poly-Universe clearly shows positive effects on the participating students throughout several studies, especially relating visual perception and attitude change toward STEAM-related tasks. It can therefore be assumed that Poly-Universe can also be usefully applied to other subjects and school levels (Dardai, 2018; Hoffmann, 2020). Based on these conclusions and preliminary results, new and adapted teaching materials with the game were invented for the three Austrian subjects, B, PE, DGE, in order to not only teach the participating secondary school students the required teaching material in a creative, innovative, fun, and game-based way, but also to promote CT.

In further considerations and conclusions it must be included, that the majority of the 80 students were girls, and there is a risk in using the participating observation method that the objectivity can suffer due to the complete participation (DeWalt et al., 1998). Previous studies showed similar findings, that game-based learning promotes enjoyment in class and it can be assumed that it positively affects students’ collaboration (Vandercruysse et al., 2012; Hamari and Koivisto, 2015; Crocco et al., 2016). However, it must be mentioned that the three classes were deliberately chosen for these three workshops (W1-3). On the one hand, because three of the authors actively teach as in-service biology, physical education, and digital education teachers in these classes. Further, the BRG Steyr is a partner institution of the research team. To reduce researchers’ bias in reporting and analyzing the data, participants were additionally observed by authors who did not create the tasks themselves and were not actively teaching in those classes. The observations and written records were then evaluated by all authors. On the other hand, because the majority of the classes studied are girls, the research team was also keen to promote interest in CT among young people, especially girls, from an early age. In addition, it should also be noted that three workshops with 80 children has no general significance. However, a positive trend towards the use of PolyUni as an educational game to promote CT in secondary school can already be seen in this study, but has to be further researched in the future.

This study surveyed which CT concepts were positively influenced and promoted in regards to PolyUni. In summary, the following concepts and skills were be promoted using the PolyUni game within all secondary school workshops: the students had to simplify and abstract the illustration of animals (W1: e.g., an eye of a spider is represented by a square shaped PolyUni tile), geometric shapes (W2: represent geometric figures in squares, triangles and circles), and dance programs (W3: one color represents one movement) (Abstraction), recognize similarities and differences between insects and spiders (W1: e.g., number of legs; insects have three-part bodies, spiders have two-part bodies), geometric tasks (W2: e.g., number of squares), and dance moves (W3) (Pattern recognition), and had to break down complex tasks into subtasks and sub-problems (W1-3: e.g., dance program consists of different parts, repetitions) (decomposition). Regarding solutions on how to generalize animals and their characteristics (W1), a geometric shape (W2), or a dance program (W3) (Generalization), the participants had to use a wide variety of color, size, or geometric form combinations and algorithms that stood for certain movements (W3: e.g., red color stands for raising your hand up), shapes (W2: only using triangle-shaped tiles), or animal characteristics (W1: square-shaped tiles are used for legs, yellow circle-shaped PolyUni elements are used for eggs; W1-3 create an algorithm for a specific shape or dance movement) (Coding, Algorithmic Thinking). The codes of the dance steps (W3) can generally be recorded and automatically reproduced by programs (automation). All of the participants had to experiment and play with the PolyUni elements (Experimentation & Tinkering), and were able to change their approaches at any time through the solutions of others, recognize mistakes and patterns of others and thus optimize their final solutions (Debugging) in all workshops (W1-3: group discussion, group work).

The majority of the children were able to present the illustrations of their tasks (e.g., animals, dance codes) in a simplified manner (Abstraction) in order to represent the most important characteristics of, for example, insects and spiders (Generalization). Furthermore, the results concerning the breaking down of complex problems also indicate that the tasks were selected to be child- and age-appropriate and that the individual elements of PolyUni obviously invite students to try them out, and enjoyably play with their peers.

It can be assumed that a great collaboration and enjoyment during a lesson can very well have a positive effect on a student’s learning success whilst using PolyUni in an educational context. Furthermore, findings suggest that PolyUni can be used successfully outside of the math curriculum, as seen in the study by Hoffmann (2020), as well in this case in biology, digital, and physical education classes. Above all, dance programming is an innovative alternative to teach and learn CT and basic IT concepts (e.g., algorithms) in a creative way. In terms of fun, there were some differences in the second workshop (PE): a possible explanation for a slightly different result in the second workshop is that the students could not see the approaches and results of the other groups very well while working out. From this finding, it can be concluded that if the focus in a teaching unit is on finding errors (de-bugging) or code (pattern) recognition in other groups, no strenuous movement exercises, such as running, should take place at the same time.

Finally, this study also surveyed how the educational game affects the learning success in the respective subjects. Overall, from the point of view of the observers, most of the students were able to achieve the previously defined teaching and learning goals for CT and digital literacy with PolyUni, especially in the biological and digital education workshops. The results indicate that also the strenuous endurance run during the exercise probably had a negative impact on the CT results (pattern recognition and incorporation). On this point, further studies with more moderate sports exercises or shorter running phases must be carried out in the future to confirm the assumptions.

## 5. Conclusion

In this research, three workshops were held at an Austrian school by implementing Poly-Universe into the courses to examine whether the game can be used successfully in biological, digital, and physical education in secondary school, to teach the required curricula, and further, promote CT at the same time. To explore these assumptions, the participating students were observed, the photos of the presented results and during W1-3 were analyzed for their correctness, and the results were recorded in a self-designed evaluation grid, which was then evaluated. Regarding the positive influence of fun on learning outcomes, the results vary between the three workshops in this study. In W2, findings indicate that the participating students who had less fun with the exercise than the rest of the students showed poorer results in the previously defined teaching and learning goals and had more issues correctly understanding, presenting, describing, and explaining new content. Further findings indicate that most groups in W1-3 had no problems understanding the game itself and were able to start the exercises without much additional help or further explanation. The results show that the tasks and explanations were designed to be age- and student-appropriate and that the students also understood the basics of PolyUni.

The majority of participants met their teaching and learning objectives. We examined not only the predefined teaching and learning goals of the curricula, but also other positive effects on students: During the lectures, most of the participants of all three workshops collaborated well with their peers. Furthermore, they enjoyed using the game during the lectures, especially whilst creating new codes for their dance programming.

All groups were able to use CT skills, such as abstraction, generalization, problem-solving, and the ability to break complex problems into subproblems, as well as using the files to tinker, play, or experiment with the PolyUni game. Additionally, all students were able to identify similarities, differences, and recognize patterns to solve complex problems. Most of the participating students used codes of geometric shapes, sizes, and color combinations, to correctly present new or previous learning biological content or to solve the physical education tasks. It is also striking that, especially in the first workshop, most of the students recognized their own mistakes in their approaches and codes and were able to solve them well by comparing them with others. Overall, from the point of view of the observers, most of the students were able to achieve the previously defined teaching and learning goals for CT PolyUni, especially in the first and last workshops.

Therefore, it can be assumed that the game is a great educational tool in various subjects in the secondary school promoting CT skills. The original learning benefit for which this game was designed can also be extended to teach the required curriculum in DGE and promote CT in B, DGE, and PE. In addition to the examples given in the article, in the “PUSE Methodology” material collection for primary and secondary school students, there are numerous teaching examples for various STEAM subjects available online as OER. These range from simple mathematical tasks (e.g., recreating given geometric shapes) up to more advanced exercises (e.g., Biology: characteristics of flowering plants).

## 6. Outlook

The participating students in this study showed increased collaboration and enjoyment during the workshops, therefore, further studies with adapted B, PE, DGE, and dance exercises in Austrian elementary and primary schools will take place in winter and summer 2023. Additional qualitative research will also take place in 2023 and 2024. In addition, further workshops for secondary education, not only for B, PE, and DGE, but also for other subjects, are planned on this promising topic in 2023.

## Data availability statement

The original contributions presented in the study are included in the article/supplementary material, further inquiries can be directed to the corresponding author/s.

## Ethics statement

Ethical review and approval was not required for the study on human participants in accordance with the local legislation and institutional requirements. Written informed consent to participate in this study was provided by the participants’ legal guardian/next of kin. Written informed consent was obtained from the minor(s)’ legal guardian/next of kin for the publication of any potentially identifiable images or data included in this article.

## Author contributions

ES, MScha, and MSchm contributed to conception, evaluation and design of the study, further organized the database and performed the statistical analysis and wrote the first draft of the manuscript. BA, BS, and ZL contributed in the conception. ES, MScha, MSchm, and SH wrote sections of the manuscript. CH and MR contributed especially in revision. All authors contributed to the article and approved the submitted version.

## Funding

This study was supported by the project Poly-Universe in Teacher Training Education, ID: KA203-DE88CF6B.

## Conflict of interest

Author SH was employed by Dynatrace.

The remaining authors declare that the research was conducted in the absence of any commercial or financial relationships that could be construed as a potential conflict of interest.

## Publisher’s note

All claims expressed in this article are solely those of the authors and do not necessarily represent those of their affiliated organizations, or those of the publisher, the editors and the reviewers. Any product that may be evaluated in this article, or claim that may be made by its manufacturer, is not guaranteed or endorsed by the publisher.

## Appendix

CTC-AG curriculum assessment grid (BMBWF, 2022a,b).

**Table:**

Group number:–––––––––––––––––– Name of class: ––––––– Date: –––––––– Number of participants: ––––––––– Age:–––––––––– Location:––––––––– Female:––––––––––––– Male:–––––––––– Name of the school: ––––––––––						
Please have the teacher tick where applicable during and after the lesson		1 Far beyond the essentials	2 Beyond the essentials	3 Fully met in the essential areas	4 Largely fulfilled in the essential areas	5 Not fulfilled in the essential areas/not rateable
	Task					
1	After the teacher has explained the game Poly-Universe, the students have understood the content of the game and its process and function					
2	After the teacher has explained the tasks, the students have understood the content of the worksheets and tasks with Poly-Universe					
3	After the teacher explained the tasks, the students could start with the Poly-Universe work tasks without additional help from the teacher					
4	The students enjoy using Poly-Universe in the lecture					
	Curriculum Biology/Physical Education/Digital Education					
5	The students can use and present previous knowledge correctly with the game Poly-Universe					
6	The students can correctly describe and explain previous knowledge with the game Poly-Universe					
7	The students can correctly understand and present new learning/teaching content with the game Poly-Universe					
8	The students can correctly describe and explain new learning/teaching content with the game Poly-Universe					
9	With the Poly-Universe game, the teacher has achieved the teaching/learning goals set in advance					
	Computational thinking					
10	The students tinker, play and/or experiment to solve the problem and task					
11	The students can break down a complex biological problem into sub-problems and thus solve the tasks					
12	Students can use Poly-Universe to identify similarities, differences, and patterns to solve complex subject-specific problems					
13	The students can hide unimportant details so that they can focus on the essential aspects of the problem and thus solve the task					
14	The students use new/identical geometric figures, shapes and combinations as codes in order to correctly present (subject-specific) content, to solve the task					
15	The students use new and the same color combinations as codes to correctly display content and subject-specific content and to solve the task					
16	The students use new and identical size combinations as codes in order to correctly display content and subject-specific content and to solve the task					
17	The students find mistakes on their own and can improve them on their own					
18	Students can recognize patterns and codes from other groups and incorporate them into their own codes and representations to improve them					
19	The students use algorithms and algorithmic thinking to correctly present content and subject-specific content and to solve the task					
20	The students work together to solve the problem and the task together					
21	With Poly-Universe, the teacher has achieved the previously defined teaching/learning goals for computational thinking and digital education					
